# Enterotype May Drive the Dietary-Associated Cardiometabolic Risk Factors

**DOI:** 10.3389/fcimb.2017.00047

**Published:** 2017-02-23

**Authors:** Ana C. F. de Moraes, Gabriel R. Fernandes, Isis T. da Silva, Bianca Almeida-Pititto, Everton P. Gomes, Alexandre da Costa Pereira, Sandra R. G. Ferreira

**Affiliations:** ^1^Department of Epidemiology, School of Public Health, University of São PauloSão Paulo, Brazil; ^2^René Rachou Research Center, Oswaldo Cruz FoundationBelo Horizonte, Brazil; ^3^Department of Preventive Medicine, Federal University of São PauloSão Paulo, Brazil; ^4^Laboratory of Genetics and Molecular Cardiology, Heart Institute (Incor), University of São Paulo Medical SchoolSão Paulo, Brazil

**Keywords:** gut microbiota, enterotype, cardiometabolic risk, diet, lipid profile

## Abstract

Analyses of typical bacterial clusters in humans named enterotypes may facilitate understanding the host differences in the cardiometabolic profile. It stills unknown whether the three previously described enterotypes were present in populations living below the equator. We examined how the identification of enterotypes could be useful to explain the dietary associations with cardiometabolic risk factors in Brazilian subjects. In this cross-sectional study, a convenience sample of 268 adults (54.2% women) reported their dietary habits and had clinical and biological samples collected. In this study, we analyzed biochemical data and metagenomics of fecal microbiota (16SrRNA sequencing, V4 region). Continuous variables were compared using ANOVA, and categorical variables using chi-square test. Vsearch clustered the operational taxonomic units, and Silva Database provided the taxonomic signatures. Spearman coefficient was used to verify the correlation between bacteria abundances within each enterotype. One hundred subjects were classified as omnivore, 102 lacto-ovo-vegetarians, and 66 strict vegetarians. We found the same structure as the three previously described enterotypes: 111 participants were assigned to *Bacteroides*, 55 to *Prevotella*, and 102 to *Ruminococcaceae* enterotype. The *Prevotella* cluster contained higher amount of strict vegetarians individuals than the other enterotypes (40.0 vs. 20.7 and 20.6, *p* = 0.04). Subjects in this enterotype had a similar anthropometric profile but a lower mean LDL-c concentration than the *Bacteroides* enterotype (96 ± 23 vs. 109 ± 32 mg/dL, *p* = 0.04). We observed significant correlations between bacterial abundances and cardiometabolic risk factors, but coefficients differed depending on the enterotype. In *Prevotella* enterotype, *Eubacterium ventriosum* (r BMI = −0.33, *p* = 0.03, and r HDL-c = 0.33, *p* = 0.04), *Akkermansia* (r 2h glucose = −0.35, *p* = 0.02), *Roseburia* (r BMI = −0.36, *p* = 0.02 and r waist = −0.36, *p* = 0.02), and *Faecalibacterium* (r insulin = −0.35, *p* = 0.02) abundances were associated to better cardiometabolic profile. The three enterotypes previously described are present in Brazilians, supporting that those bacterial clusters are not population-specific. Diet-independent lower LDL-c levels in subjects from *Prevotella* than in other enterotypes suggest that a protective bacterial cluster in the former should be driving this association. Enterotypes seem to be useful to understand the impact of daily diet exposure on cardiometabolic risk factors. Prospective studies are needed to confirm their utility for predicting phenotypes in humans.

## Introduction

Cardiometabolic diseases are among the leading causes of mortality, and an unhealthy diet plays a significant etiopathogenetic role (World Health Organization, 2011; Laslett et al., 2012). Pieces of evidence indicate that the gut microbiota mediates the relationship between dietary habits and cardiometabolic abnormalities (Koeth et al., 2013; Yin et al., 2015). The vast number of intestinal bacteria, and the large intra- and inter-individual variability has limited the understanding of such relationship. The observation of bacterial clusters in human gut has represented a way to reduce the complexity of these analyses. Arumugam et al. (2011) found three bacteria groups in humans, driven by high proportions of one of three taxa: *Bacteroides* (enterotype 1), *Prevotella* (enterotype 2), and *Ruminococcus* (enterotype 3). The bacterial communities play an important role driving diverse pathophysiological processes (Arumugam et al., 2011). Another study discussed the associations of dietary habits with two enterotypes, distinct from this seminal study since the *Bacteroides* enterotype was fused with the less distinct *Ruminococcus* enterotype (Wu et al., 2011). Animal protein and fat intake were associated with *Bacteroides* cluster, while *Prevotella* with a carbohydrate-enriched diet.

Populations are exposed to different dietary habits, and it is unknown how the enterotypes are distributed worldwide. Most studies that describe the enterotypes involves European, North American, and Asian (Arumugam et al., 2011; Wu et al., 2011; Lim et al., 2014; Roager et al., 2014) populations. Only a few scientific publications analyzed the clusters in South American or African individuals (Yatsunenko et al., 2012; Ou et al., 2013). The knowledge on the distribution of enterotypes, in populations with different genetic backgrounds and lifestyle, could be useful to understand underlying mechanisms linking dietary habits with the risk of cardiometabolic diseases (Zupancic et al., 2012; Koeth et al., 2013; Kelder et al., 2014).

Brazilian population offers an opportunity to investigate the presence of enterotypes in a high-food variety environment, and to deepen knowledge on the role of the gut microbiota mediating the impact of diet on metabolic disturbances. We hypothesized that enterotypes might participate in underlying mechanisms linking dietary habits to cardiometabolic diseases. We investigated whether enterotypes could be identified in a sample of Brazilians and examined the impact of this categorization of the gut microbiota on the association with the cardiometabolic profile.

## Materials and methods

### Subjects

In this cross-sectional analysis, we included a convenience sample of 268 participants from the major study named ADVENTO—Analysis of Diet and Lifestyle for Cardiovascular Prevention in Seventh-Day Adventists (http://www.estudoadvento.org). The ethical committee of the School of Public Health, Univesity of São Paulo, approved this study; all individuals provided written consent. Inclusion criteria were age from 35 to 65 years and body mass index (BMI) <40 kg/m2. Diabetes mellitus, history of inflammatory bowel diseases, persistent diarrhea, and use of antibiotics or probiotic or prebiotic supplements within the 2 months before the data collection were exclusion criteria. Dietary data was obtained using a validated food frequency questionnaire from the ADVENTO. Subjects were classified according to the dietary habit adopted for at least 12 months, in strict vegetarian (no consumption of animal products), lacto-ovo-vegetarian (consumption of dairy products and/or eggs), and omnivore (consumption of animal products more than once a month; Tonstad et al., 2009).

### Clinical data

Weight was measured using a digital scale with 200 kg capacity, height using a fixed stadiometer and BMI was calculated as weight in kilograms divided by height in meters squared. Blood pressure (BP) was measured with a standard oscillometric device (Omron HEM 705CPINT, Omron Health Care, Lake Forest, IL, USA). Blood samples were taken after an overnight fasting. Plasma glucose was measured by the hexokinase method (ADVIA Chemistry; Siemens, Deerfield, IL, USA). Measurements of total cholesterol, triglyceride, and high-density lipoprotein (HDL-c) were assessed by enzymatic methods. Low-density lipoprotein cholesterol (LDL-c) was calculated by the Friedewald equation.

### Gut microbiota

The analysis of the 16S rRNA gene (V4 region) was performed by Illumina® MiSeq platform using 200 mg of fecal samples maintained under refrigeration (6°C) within a maximum of 24 h after collection, and the aliquots stored at −80°C until analysis. The Maxwell® 16 DNA purification kit was used to extract DNA, and the manufacturer's instruction was used to carry out the protocol in the Maxwell® 16 Instrument (Promega, Madison, WI, USA). The DNA was amplified by a PCR assay using the 515F and 806R primers, as described by Caporaso et al. (2012), and sequenced in Illumina Miseq platform generating paired reads of 250 bp. 16S ribosomal DNA sequences are available under study accession PRJEB19103.

The paired reads were trimmed to remove bases with Phred score lower than five at the 5′ and 3′ extremities. These procedures also trimmed sequences with an average quality <15 in a sliding window of 4 bases. The software Trimmomatic (Bolger et al., 2014) performed this quality filter. Paired reads were merged using the FLASh tool (Magoč and Salzberg, 2011), requiring a minimum overlap of 20 nucleotides.

The redundancy among the sequences was removed using the dereplication step from Vsearch (Rognes et al., 2016), and filtered to remove the unique entries. The dereplicated reads with 97% identity were clustered, using the same tool, to create the OTUs. Taxonomical assignment to the OTUs was performed by the assign_taxonomy script from Qiime (Caporaso et al., 2010) and Silva database, version 123 (Quast et al., 2013).

### Enterotype clustering

The enterotypes were identified by the methods previously described (Arumugam et al., 2011, 2014) and available in http://enterotype.embl.de/enterotypes.html. The Calinski-Harabasz (CH) index suggested the optimal number of clusters. A silhouette analysis and elbow plot evaluated the groups' robustness (Supplementary Figure S1).

### Statistical analysis

The descriptive statistical analysis calculated means, standard deviations, medians, and interquartile ranges. Variables with skewed distributions were log-transformed before analysis to achieve normality. ANOVA with Bonferroni *post-hoc* test was used to compare variables according to enterotypes and diet. Chi-square test was employed to compare proportions. The Spearman correlation coefficient pointed associations between metadata and most common genera or species (present in at least 80% of subjects). The most abundant genera were shown in the figures. Statistical analyses were performed using Statistical Package for the Social Sciences (SPSS), version 23 (IBM, Armonk, NY, USA), and R for enterotype analyses (cluster package). Beta diversity comparisons were computed as Principal Coordinate Analyses generated from Jensen-Shannon divergence matrices. A *p* < 0.05 was considered to identify important correlations.

## Results

The mean age of participants was 49.4 ± 8.4 years, 54.2% were women and 41.4% and had increased BMI (≥25 kg/m2). Sixty-six subjects were considered strict vegetarians, 102 lacto-ovo-vegetarians, and 100 omnivores. Strict and lacto-ovo-vegetarians had lower BMI (23.1 ± 4.1 and 24.4 ± 3.9 vs. 26.4 ± 4.7 kg/m2, respectively, *p* < 0.001) and LDL-c values (99 ± 31 and 101 ± 27 vs. 112 ± 29 mg/dL, respectively, *p* = 0.005) than omnivores (Supplementary Table S1).

**Figure 1:**
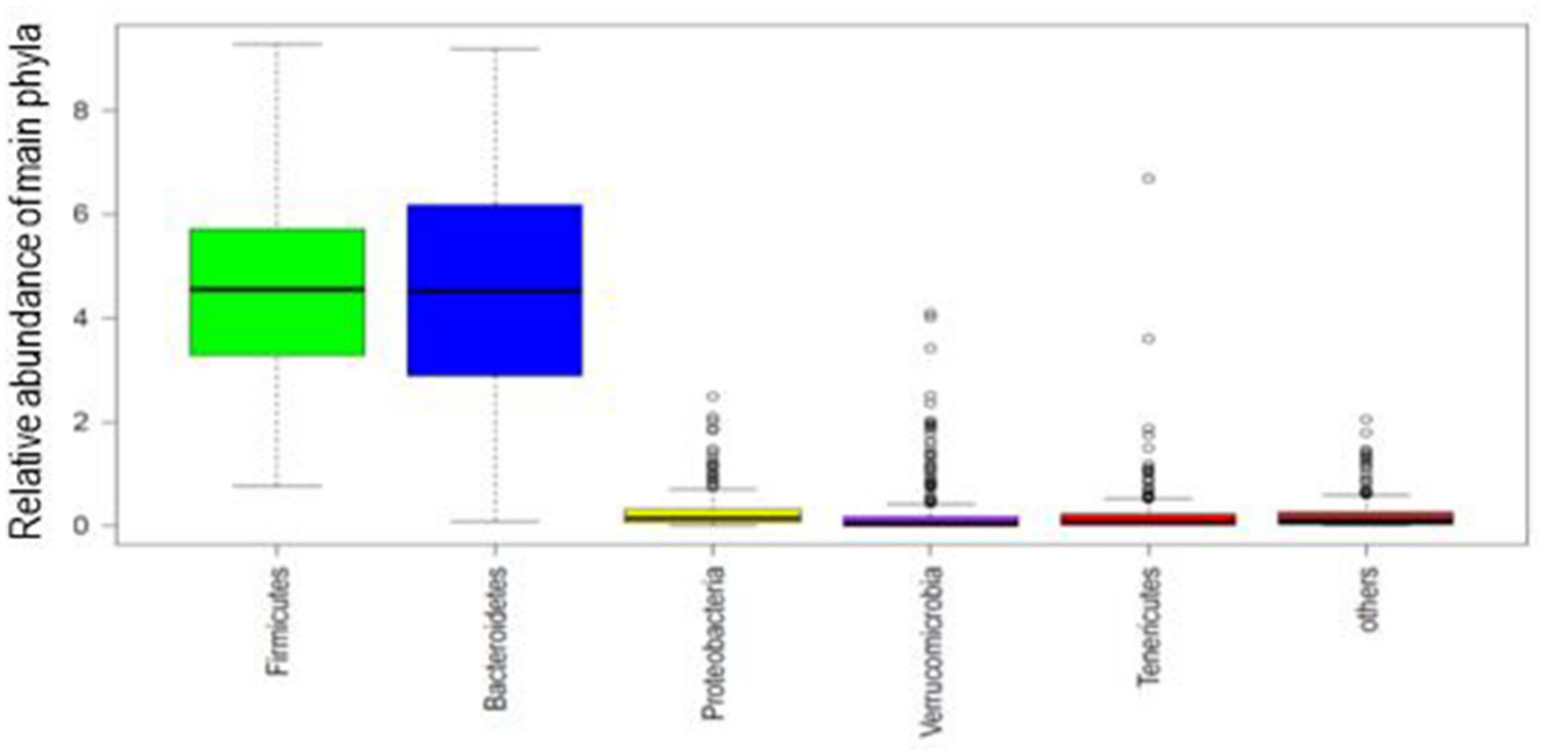
**Box plot of the phylogenetic profile of the fecal microbiota of 268 participants according to phyla abundance**. The five most abundant phyla are shown and the rest as others. Boxes represent the interquartile range and the line inside represents the median.

Taxonomical distribution of fecal samples showed the predominance of *Firmicutes* and *Bacteroidetes* (Figure 1). The 10 most abundant phyla and 20 genera according to enterotypes and dietary habits were depicted in Supplementary Figure S2.

**Figure 2:**
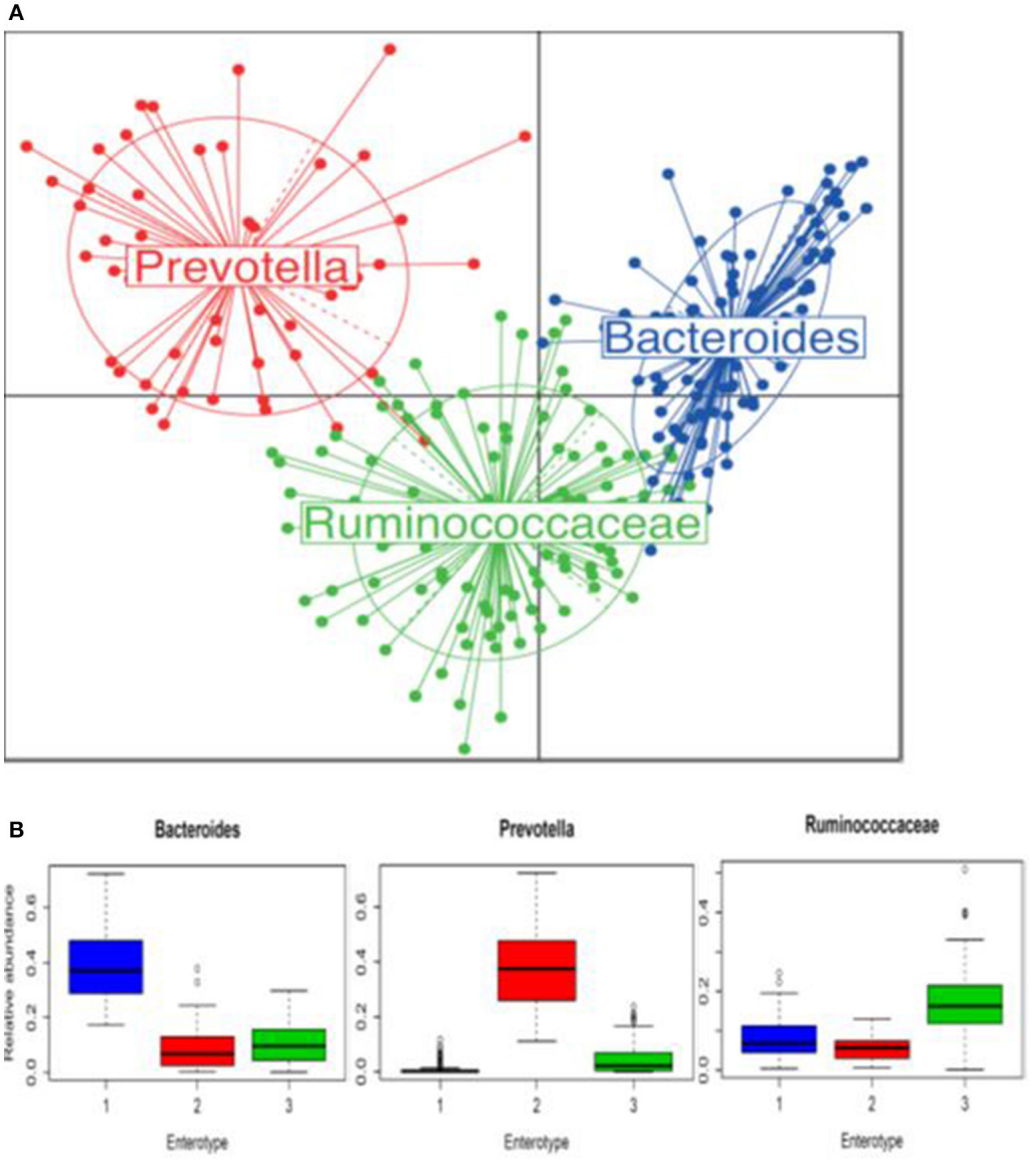
**Enterotypes identified in 268 participants using Principal Coordinate Analysis. (A)** Samples colored by the enterotype they belong to: blue is enterotype 1 (*Bacteroides*), red is enterotype 2 (*Prevotella*) and green is enterotype 3 (*Ruminococcaceae*). **(B)** Relative abundances of *Bacteroides, Prevotella*, and *Ruminococcaceae* in each enterotype. Boxes represent the interquartile range and the line inside represents the median.

Three bacterial clusters were identified; 111 participants were assigned to *Bacteroides*, 55 to *Prevotella*, and 102 to *Ruminococcaceae* enterotype (Figure 2A). Relative abundances in each enterotype confirmed the expected predominance of genera *Bacteroides, Prevotella*, and *Ruminococcaceae*, respectively (Figure 2B). Subjects in each enterotype did not differ according to sex distribution, mean age, and BMI. The frequency of strict vegetarians was greater in *Prevotella* than in the *Bacteroides* and *Ruminococacceae* enterotypes (40.0 vs. 20.7 and 20.6%, *p* = 0.04, respectively), but frequencies of lacto-ovo-vegetarians and omnivores did not differ (Supplementary Figure S3).

Comparisons of clinical variables among enterotypes showed lower mean LDL-c values in *Prevotella* compared to *Bacteroides* (96 ± 23 vs. 109 ± 32 mg/dL, *p* = 0.04), despite similar measurements of body adiposity (Table 1). When a sub-stratification of Supplementary Table S1 comparing enterotypes according to dietary habits (Supplementary Table S2), the lowest LDL-c levels were invariably observed in the *Prevotella* enterotype independently of the dietary pattern. Within the *Prevotella* enterotype, the strict vegetarian and lacto-ovo-vegetarian showed mean LDL-c values significantly lower than omnivores (92 ± 23 and 88 ± 18 vs. 107 ± 25 mg/dL, respectively, *p* = 0.04). Strict vegetarians belonging to the *Ruminococcaceae* cluster had the greatest mean value of HDL-c that was significantly higher than subjects from the same enterotype but consumers of other dietary habits (59 ± 1 and 47 ± 1 vs. 51 ± 1 mg/dL, respectively, *p* = 0.004).

**Table 1:** **Mean values (±standard deviation) of clinical and biochemical data of 268 participants according to their enterotypes**.

	***Bacteroides n*** **= 111**	***Prevotella n*** **= 55**	***Ruminococcaceae n*** **= 102**	***P*****-value**
Body mass index (kg/m^2^)	25.1 ± 4.6	24.5 ± 4.2	24.8 ± 4.5	0.743
Waist circumference (cm)	83.2 ± 12.0	83.4 ± 10.8	82.5 ± 12.5	0.886
Systolic BP (mmHg)	115 ± 15	120 ± 12	116 ± 14	0.060
Diastolic BP (mmHg)	72 ± 10	75 ± 8	72 ± 10	0.184
Plasma glucose (mg/dL)	93 ± 8	94 ± 12	92 ± 7	0.337
Fasting insulin^#^ (μUI /mL)	8.4 ± 1.7	7.3 ± 1.9	7.5 ± 1.7	0.205
Total cholesterol (mg/dL)	181 ± 40	169 ± 25	179 ± 35	0.093
LDL-cholesterol (mg/dL)	109 ± 32	96 ± 23^Ω^	105 ± 29	0.036
HDL-cholesterol^#^ (mg/dL)	50 ± 1	49 ± 1	51 ± 1	0.648
Triglycerides^#^ (mg/dL)	93 ± 2	100 ± 1	91 ± 2	0.458

*BP, blood pressure*.

# *Log-transformed values for analysis and were back-transformed to return to the natural scale. ANOVA followed by Bonferroni post hoc test*.

Ω *vs. Bacteroides*.

**Figure 3:**
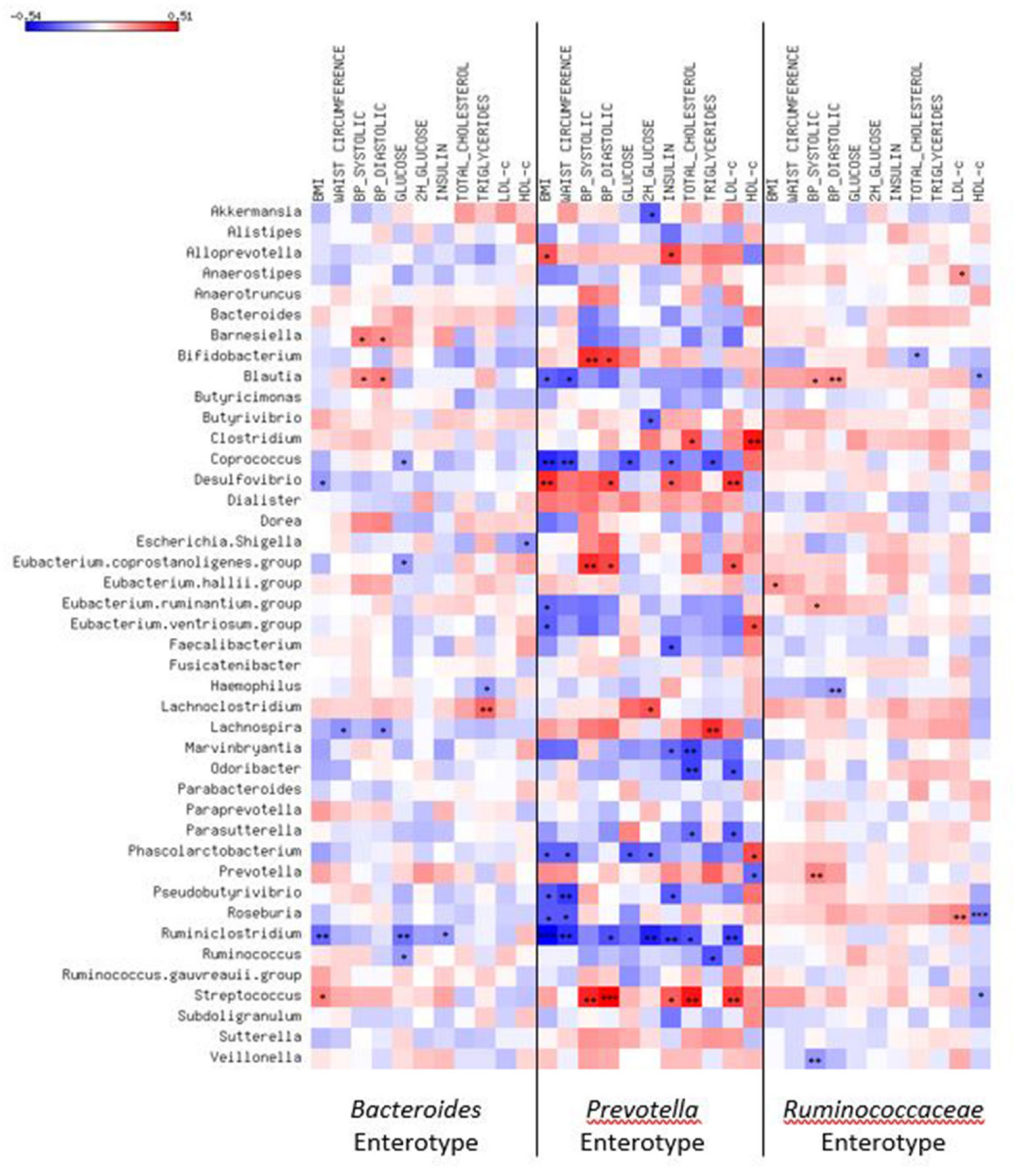
**Heatmap of correlations of most common genera or species and cardiometabolic risk factors according to enterotypes**. Spearman correlation test used. ^*^p < 0.05; ^**^p < 0.01; ^***^p < 0.001; BMI, body mass index; BP, blood pressure.

Correlations of clinical variables to bacteria abundances considering the entire sample ranged from −0.23 to 0.21. When stratified by enterotypes (Figure 3), the coefficients changed. In *Bacteroides* cluster, the abundance of *Streptococcus* was correlated to body adiposity (r BMI = 0.25, *p* = 0.02) and *Blautia* to systolic (*r* = 0.22, *p* = 0.04) and diastolic BP (*r* = 0.26, *p* = 0.01), while abundances of *Desulfovibrio* were inversely correlated to BMI (*r* = −0.22, *p* = 0.04) and *Haemophilus* to triglyceride levels (*r* = −0.22, *p* = 0.04).

The strongest correlations coefficients to cardiometabolic risk factors were detected in the *Prevotella* enterotype. *Blautia* (r BMI = −0.34, *p* = 0.03 and r waist = −0.37, *p* = 0.02), *Coprococcus* (r BMI = −0.45, *p* < 0.01; r waist = −0.41, *p* < 0.01; r glucose = −0.37, *p* = 0.02; r insulin = −0.31, *p* = 0.04, and r triglyceride = −0.37, *p* = 0.02), *Roseburia* (r BMI = −0.36, *p* = 0.02 and r waist = −0.36, *p* = 0.02), *Faecalibacterium* (r insulin = −0.35, *p* = 0.02), *Eubacterium ventriosum* (r BMI = −0.33, *p* = 0.03 and r HDL-c = 0.33, *p* = 0.04), and *Akkermansia* (r 2h glucose = −0.35, *p* = 0.02) abundances were correlated to a better cardiometabolic profile, while *Streptococcus* (r systolic BP = 0.44, *p* = 0.004, r diastolic BP = 0.51, *p* < 0.001, r insulin = 0.33, *p* = 0.04, and r LDL-c = 0.40, *p* = 0.009) and *Desulfovibrio* (r BMI = 0.42, *p* = 0.006, r diastolic BP = 0.37, *p* = 0.02, r insulin = 0.32, *p* = 0.04, and r LDL-c = 0.40, *p* = 0.01) abundances to a worse profile.

In *Ruminococcaceae* enterotype, *Blautia* abundance was directly correlated to systolic (*r* = 0.20, *p* = 0.02) and diastolic BP (*r* = 0.22, *p* = 0.008) and inversely to HDL-c levels (*r* = −0.20, *p* = 0.02). *Roseburia* was correlated to unfavorable lipid profile (r LDL-c = 0.24, *p* < 0.01 and r HDL-c = −0.28, *p* < 0.001) and *Eubacterium hallii* to BMI (*r* = 0.21, *p* = 0.02), while *Bifidobacterium* (r total cholesterol = −0.21, *p* = 0.01) and *Haemophilus* (r diastolic BP = −0.23, *p* < 0.01) to better cardiometabolic parameters.

## Discussion

The three enterotypes, described in populations from the North hemisphere, were found in the Brazilian population in a similar structure as previously described. Our observation of increased proportion of strict vegetarians in the *Prevotella* enterotype supports that dietary habits are important determinants of commensal bacteria clustering. Additionally, vegetarian diet associated with lower LDL-c levels suggest that the presence of a protective bacterial cluster in this enterotype could be driving this association. Such consistency of findings in the *Prevotella* cluster was not seen in the other enterotypes, in which we observed different correlations between bacterial abundances and cardiometabolic parameters. A broader variety of the dietary components of the subjects from the *Bacteroides* and *Ruminococcaceae* enterotypes could have limited identifying the relationship between bacteria and risk factors.

The main phyla, *Firmicutes* and *Bacteroidetes*, as well as the most common commensal genera that usually dominate the human gut microbiota, were observed in our sample. Cluster analyses clearly identified the three bacterial groups previously described (Arumugam et al., 2011). Other studies conducted in American, Korean, and Danish populations failed to demonstrate them (Wu et al., 2011; Lim et al., 2014; Roager et al., 2014), which may be attributed, in parts, to differences in methodological approaches to clustering data (Koren et al., 2013).

An opportunist characteristic of our sample was the diversity of dietary patterns, which allowed investigating how the participants were distributed among the bacterial clusters. The diversity facilitated our interpretation of possible physiological roles of bacteria present in the enterotypes. Our findings suggest that different diet-dependent combinations of bacteria should result in different effects on the cardiometabolic profile. Apparently, the importance of genetic factors, breastfeeding, and other early life events for the cardiometabolic risk cannot be neglected.

Lower LDL-c levels were found in subjects belonging to *Prevotella* enterotype, which is consonant with the greater number of strict vegetarians in this enterotype. We speculated that the absence of animal food-derived saturated fatty acids could account for this result (Bradbury et al., 2014; Le and Sabaté, 2014), although our methods are unable to confirm such assumption. Participants classified in this enterotype were not leaner or had lower plasma glucose levels, as previously reported in subjects consuming plant-based diet (Le and Sabaté, 2014; Sabaté and Wien, 2015). Few studies examined the association of enterotypes with cardiometabolic risk factors (Zupancic et al., 2012; Lim et al., 2014); one conducted in Koreans reported increased uric acid concentration in *Bacteroides* cluster compared to the other enterotypes (Lim et al., 2014). As far as we know, this is the first study that detects differences in lipid metabolism using bacterial clustering. Furthermore, when consumers of diverse diets were stratified according to enterotypes, the lowest LDL-c values seen in *Prevotella* enterotype seems to be independent of the dietary habit. This finding suggests that bacteria associated with *Prevotella* may be important drivers of the effect in lipid metabolism.

We tested the correlations of bacteria with cardiometabolic variables within each enterotype to clarify the pathophysiological relationship. We found diverging relationships between a given genus and metabolic parameter when compared one enterotype to another. *Blautia* abundance was favorably correlated to anthropometric measurement in *Prevotella* cluster, but in *Bacteroides* and *Ruminococcaceae* enterotypes showed an unfavorable relationship with cardiometabolic parameters. This genus belonging to *Lachnospiraceae* family was more commonly found in animals consuming herbivore diet and is known due to its capacity to degrade complex polysaccharides to short-chain fatty acids, such as butyrate, acetate, and propionate (Furet et al., 2009; Biddle et al., 2013; Eren et al., 2015). Several fermentation-dependent metabolic benefits have been described. Butyrate stimulates enteroendocrine cells to secrete incretins (Kasubuchi et al., 2015; Woting and Blaut, 2016), inhibits of pro-inflammatory cytokines production (Miquel et al., 2013; Hippe et al., 2016) and enhances expression of tight-junction proteins (Cani et al., 2009; Peng et al., 2009) that improve gut barrier and reduce metabolic endotoxemia. Such effects have a protective impact on obesity and insulin resistance (Cani et al., 2009; Brahe et al., 2015; Kasubuchi et al., 2015; Hippe et al., 2016). Our findings suggest that this could be occurring in subjects belonging to the *Prevotella* enterotype. Since a high number of vegetarians was present in this enterotype, we suggest that butyrate-producing bacteria should contribute inducing several metabolic benefits.

Only in *Prevotella* enterotype, abundances of other butyrate-producing bacteria, *E. ventriosum, Roseburia, Coprococcus*, and *Faecalibacterium* (Barcenilla et al., 2000; Pryde et al., 2002; Brahe et al., 2015), showed correlations that are suggestive of a protective role of increased body adiposity and metabolic disturbances. Interestingly, the positive relationship between *E. ventriosum* abundance and HDL-c had not been described. This correlation was not an unexpected finding since another butyrate property is the capacity of activating the GPR109A, which in turn regulates lipid homeostasis (Elangovan et al., 2014). Also, this effect is coherent with lower LDL-c levels observed in participants belonging to *Prevotella* enterotype. *Coprococcus* was previously associated with adequate bacterial richness in healthy lean adults (Furet et al., 2010) and high abundance of *Faecalibacterium* in subjects consuming fiber-enriched diets (Canani et al., 2011; Matijašić et al., 2014) and low in those with obesity and type 2 diabetes (Furet et al., 2010; Zhang et al., 2013). Our findings are in agreement with the majority of investigators who suggested that these genera abundances are markers of gut health (Miquel et al., 2013; Martín et al., 2015; Hippe et al., 2016), but not all (Balamurugan et al., 2010; Feng et al., 2014). Additionally, the correlation of *Akkermansia* abundance and plasma glucose is consistent with previously reported benefits of this genus in inflammatory status and glucose metabolism (Everard et al., 2013; Schneeberger et al., 2015; Greer et al., 2016).

We speculate that the enterotype-mediated risk pattern is dependent of the local microenvironment, and the combination of abundant bacteria in each enterotype would drive the pathophysiological outcomes. The fiber-rich diet of vegetarians included in the *Prevotella* enterotype could have triggered beneficial effects at the intestinal and systemic levels. Therefore, our findings are consistent reports of favorable cardiometabolic risk profile in subjects consuming diets rich in fruits and vegetables like the Adventists (Pettersen et al., 2012; Sabaté and Wien, 2015).

Interestingly, in *Ruminococacceae* enterotype, *Eubacterium hallii*, and *Roseburia* were unfavorably associated with metabolic parameters, while *Desulfovibrio* and *Haemophilus*, from the *Proteobacteria* phylum, with a protective relationship. It is well-known that the latter are gram-negative bacteria with lipopolysaccharide on its surface. This endotoxin is an important ligand for toll-like receptor 4 that activates the innate immune system, which could result in a pro-inflammatory condition (Cani et al., 2009; Velloso et al., 2015). Considering that *Proteobacteria* preferentially metabolize proteins (Ferrocino et al., 2015), higher abundance of bacteria from this phylum could be expected in *Bacteroides* and *Ruminococacceae* enterotypes, in which lacto-ovo-vegetarians and omnivores were more commonly present. This agrees with a report of high abundance of *Proteobacteria* in children consuming a protein-fat based diet (De Filippo et al., 2010). However, some inverse correlations with cardiometabolic factors were unexpectedly detected in both enterotypes. Only in the *Prevotella* enterotype, *Desulfovibrio* abundance was directly correlated to BMI, BP, insulin, and LDL-c, in line with previous animal and human studies. In db/db mice (Geurts et al., 2011) and humans with cardiovascular diseases (Yin et al., 2015) compared to respective controls, *Proteobacteria* was more abundant. Such results may reinforce that the resulting balance of a great variety of bacteria present in gut drives metabolic processes in the host. Therefore, different diet-dependent combinations of bacteria would be related to distinct cardiometabolic risk profile. Comparisons of clinical data of subjects within each diet stratified by enterotype reinforced our assumption that enterotype may be driving the dietary lipid-associated risk since the LDL-c values were invariably lower in the subsets of participants from the *Prevotella* enterotype.

In all enterotypes, the abundance of *Streptococcus* was correlated to unfavorable cardiometabolic risk profile (increased adiposity, BP, and lipids), although correlation coefficients in the *Ruminococcaceae* enterotype were weak (data not shown). This genus belongs to *Firmicutes* phylum, which was originally described as the predominant in animal obesity (Ley et al., 2005; Turnbaugh et al., 2006). We have reported a greater abundance of *Streptococcus alactolyticus* in obese animals compared to hypertensive and Winstar rat (Petriz et al., 2014). Our correlations might be in part due to its proinflammatory role previously described (Al-Jashamy et al., 2010; Jiang et al., 2015).

Our study has limitations. Regarding the dietary intake assessment, raw data were not available impeding to establish associations of nutrients and the microbiota. Determination of fecal supernatants would be desirable to support the assumption of a lower content of fat among strict vegetarian subjects. Fecal consistency was not systematically obtained and was not considered as a confounder in our analyses. Recently, the influence of fecal consistency with gut microbiota richness and composition and bacterial growth rates has been raised (Vandeputte et al., 2016). Our sample from the ADVENTO study is not representative of the general population living in Brazil. As a matter of fact, smoking and drinking habits are known to be less frequent among Adventists. On the other hand, such characteristics should have contributed to minimizing confounders in our analyses.

In conclusion, the three enterotypes previously described are present in Brazilians, supporting that those bacterial clusters are not population-specific. Diet-independent lower LDL-c levels in subjects from *Prevotella* than in other enterotypes suggest that a protective bacterial group in the former should be driving this association. Enterotypes seem to be useful to understand the impact of daily diet exposure on cardiometabolic risk factors. Prospective studies are needed to confirm their utility for predicting phenotypes in humans.

## Author contributions

Ad, BA, SF had substantial contributions to the conception and design of the work. Ad, EG, AP, SF to the acquisition of data. Ad, GF, Id, SF to the analysis and interpretation of data for the work. Ad, Id, SF drafted the work and GF, BP, EG, AP revised it critically for important intellectual content. Ad, GF, Id, BP, EG, AP, SF participated of the final approval of the version to be published. Ad, GF, Id, BP, EG, AP, SF agreed to be accountable for all aspects of the work in ensuring that questions related to the accuracy or integrity of any part of the work are appropriately investigated and resolved.

## Funding

The present study was supported by FAPESP (2012/12626-9 and 2012/03880-9).

### Conflict of interest statement

The authors declare that the research was conducted in the absence of any commercial or financial relationships that could be construed as a potential conflict of interest.
