# Supplementary material for: Enterotype May Drive the Dietary-Associated Cardiometabolic Risk Factors
Source: Front Cell Infect Microbiol. 2017 Feb 23;7:47. doi: 10.3389/fcimb.2017.00047 (PMC5322172; doi:10.3389/fcimb.2017.00047)
Supplement: Supplementary file 1 [file Table1.PDF]

## *Supplementary Material*

# Enterotype may drive the diet-associated cardiometabolic risk factor

**Ana Carolina Franco de Moraes, Gabriel R. Fernandes, Isis Tande da Silva, Bianca Almeida-Pititto, Everton Padilha Gomes, Alexandre da Costa Pereira, Sandra Roberta G. Ferreira*.**

*** Correspondence:** Corresponding Author: sandrafv@usp.br

## 1 Supplementary Figures and Tables

### 1.2 Supplementary Tables

**Supplementary Table S1. Mean values (± standard deviation) of clinical and biochemical data of 268 participants according to their dietary habits.**

| | Strict vegetarian n = 66 | Lacto-ovo-vegetarian n = 102 | Omnivore n = 100 | *P*-value |
|---|---|---|---|---|
| Body mass index (kg/m$^2$) | 23.1 ± 4.1 | 24.4 ± 3.9 $^{\Omega}$ | 26.4 ± 4.7 $^{\Omega¥}$ | **<0.001** |
| Mean blood pressure (mmHg) | 87 ± 11 | 88 ± 12 | 89 ± 11 | 0.403 |
| Plasma glucose (mg/dL) | 92 ± 8 | 92 ± 7 | 95 ± 10 | 0.076 |
| Total cholesterol (mg/dL) | 174 ± 37 | 173 ± 36 | 185 ± 34 $^{¥}$ | **0.028** |
| LDL-cholesterol (mg/dL) | 99 ± 31 | 101 ± 27 $^{\Omega}$ | 112 ± 29 $^{\Omega¥}$ | **0.005** |
| Non HDL-cholesterol (mg/dL) | 120 ± 36 | 121 ± 31 $^{\Omega}$ | 134 ± 34 $^{\Omega¥}$ | **0.012** |

ANOVA followed by Bonferroni post hoc test
$^{\Omega}$ *versus* strict vegetarian  $^{¥}$ *versus* lacto-ovo-vegetarian
