# Supplementary material for: Enterotype May Drive the Dietary-Associated Cardiometabolic Risk Factors
Source: Front Cell Infect Microbiol. 2017 Feb 23;7:47. doi: 10.3389/fcimb.2017.00047 (PMC5322172; doi:10.3389/fcimb.2017.00047)
Supplement: Supplementary file 2 [file Table2.pdf]

Supplementary Material

Enterotype may drive the diet-associated cardiometabolic risk factor

Ana Carolina Franco de Moraes, Gabriel R. Fernandes, Isis Tande da Silva, Bianca Almeida-Pititto, Everton Padilha Gomes, Alexandre da Costa Pereira, Sandra Roberta G. Ferreira*.

*** Correspondence:** Corresponding Author: sandrafv@usp.br

1 Supplementary Figures and Tables

1.2 Supplementary Tables

Supplementary Table S2. Mean values (± standard deviation) of clinical and biochemical data of 268 participants stratified by their dietary habits and enterotypes.

	Strict vegetarian				Ovo-lacto-vegetarian				Omnivore				*P*	*P*	*P*
	ET1	ET2	ET3	*P*	ET1	ET2	ET3	*P*	ET1	ET2	ET3	*P*	ET1	ET2	ET3
BMI (kg/m^2)	23.1±3.9	23.5±4.0	23.0±4.8	0.920	24.8±4.6	24.4±3.3	23.8±3.2	0.469	26.5±4.7	25.8±4.8	26.7±4.8	0.801	**0.018**	0.214	**0.001**
Waist (cm)	81±12	81±9	77±11	0.392	82±12	84±9	80±10	0.481	88±12	85±13	87±14	0.817	0.190	0.388	**0.002**
SystBP (mmHg)	116±18	117±11	112±13	0.402	116±15	126±18	117±15	0.120	117±15	124±13	118±14	0.206	0.989	0.139	0.253
DiastBP (mmHg)	73±12	74±8	70±7	0.358	72±9	77±9	73±12	0.329	73±11	77±8	74±10	0.350	0.902	0.298	0.361
Glucose (mg/dL)	91±9	92±8	93±7	0.795	93±7	93±6	91±8	0.460	94±9	98±16	93±7	0.212	0.313	0.211	0.456
Insulin$^{\#}$ (µUI /mL)	6.0±1.6	7.1±1.9	6.5±1.7	0.630	8.4±1.6	6.6±1.8	6.6±1.7	0.070	10.0±1.7	8.2±1.8	8.9±1.8	0.380	**0.001**	0.578	**0.028**
Total-c (mg/dL)	174±38	164±24	184±45	0.232	178±42	161±20	171±31	0.296	190±37	180±27	183±33	0.481	0.218	0.069	0.233
LDL-c (mg/dL)	103±32	92±23	103±37	0.439	104±30	88±18	101±25	0.145	117±33	107±25	110±27	0.344	0.103	**0.042**	0.375
HDL-c$^{\#}$ (mg/dL)	49±1	48±1	59±1$^{\Omega¥}$	**0.007**	52±1	50±1	47±1	0.351	49±1	51±1	51±1	0.662	0.626	0.620	**0.004**
Triglyc$^{\#}$ (mg/dL)	91±2	112±1	88±2	0.158	91±2	98±1	92±2	0.848	96±2	91±2	92±2	0.888	0.841	0.252	0.943

ET1, enterotype 1 – *Bacteroides*. ET2, enterotype 2 – *Prevotella*. ET3, enterotype 3 – *Ruminococcaceae*. BP, blood pressure. Triglyc, triglycerides. *P*, *P*-value. $^{\#}$ Log-transformed values for analysis and were back-transformed to return to the natural scale. ANOVA followed by Bonferroni post hoc test. $^{\Omega}$ versus *Bacteroides* $^{¥}$ *versus* to *Ruminococcaceae.*
