# Supplementary material for: Enterotype May Drive the Dietary-Associated Cardiometabolic Risk Factors
Source: Front Cell Infect Microbiol. 2017 Feb 23;7:47. doi: 10.3389/fcimb.2017.00047 (PMC5322172; doi:10.3389/fcimb.2017.00047)
Supplement: Supplementary file 3 [file Image1.PDF]

Supplementary Material

Enterotype may drive the diet-associated cardiometabolic risk factor

Ana Carolina Franco de Moraes, Gabriel R. Fernandes, Isis Tande da Silva, Bianca Almeida-Pititto, Everton Padilha Gomes, Alexandre da Costa Pereira, Sandra Roberta G. Ferreira*.

*** Correspondence:** Corresponding Author: sandrafv@usp.br

1 Supplementary Figures and Tables

1.1 Supplementary Figures

(A)

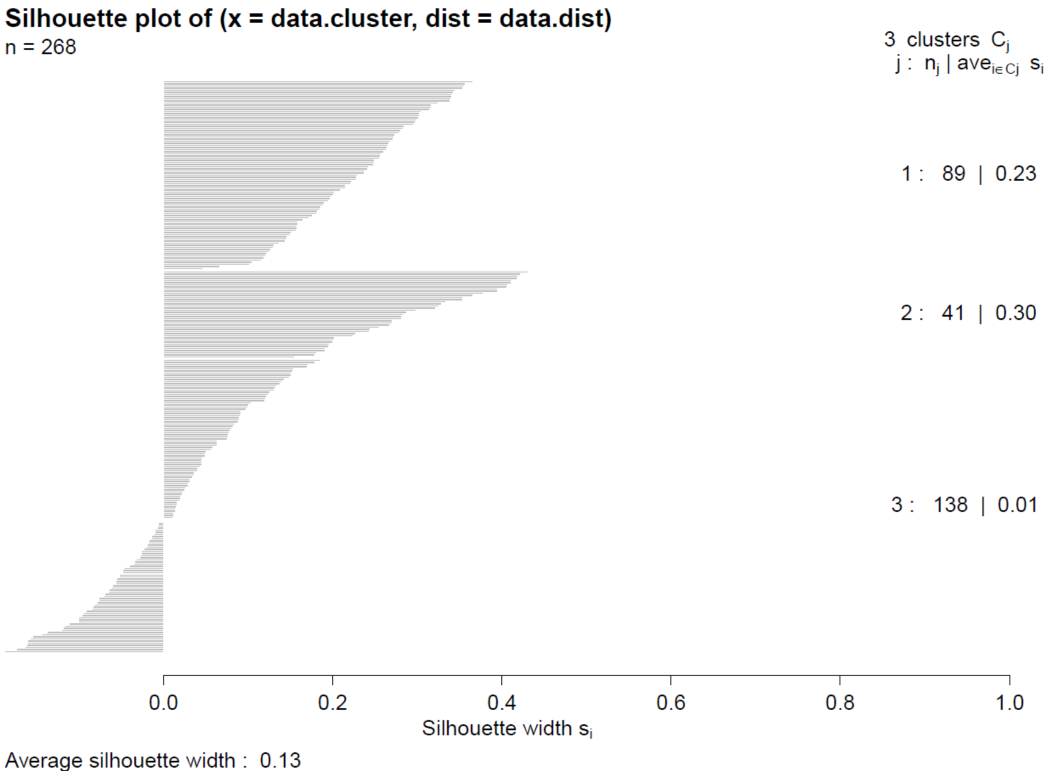

(B)

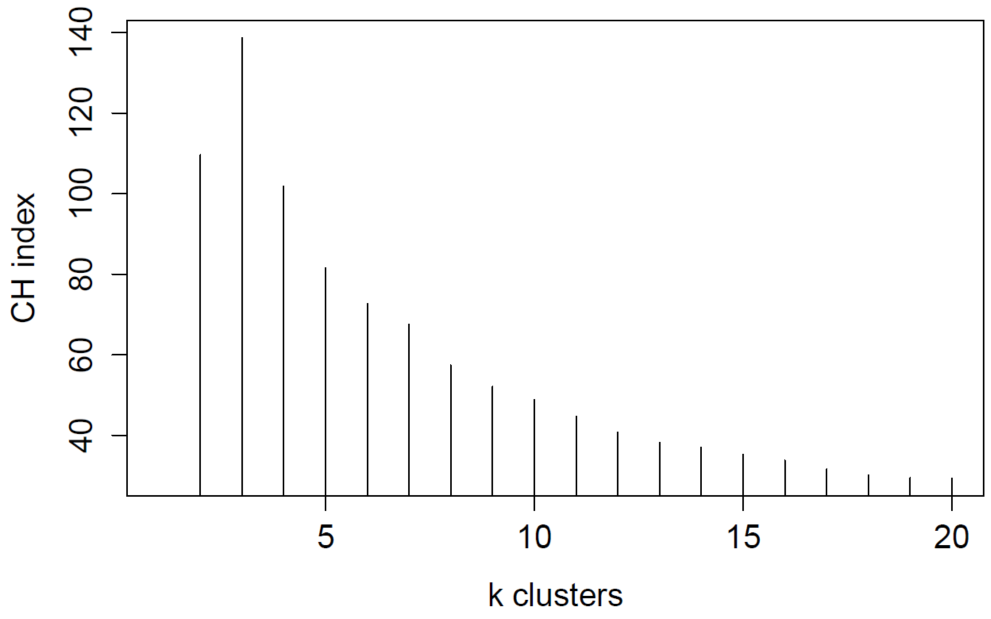

(C)

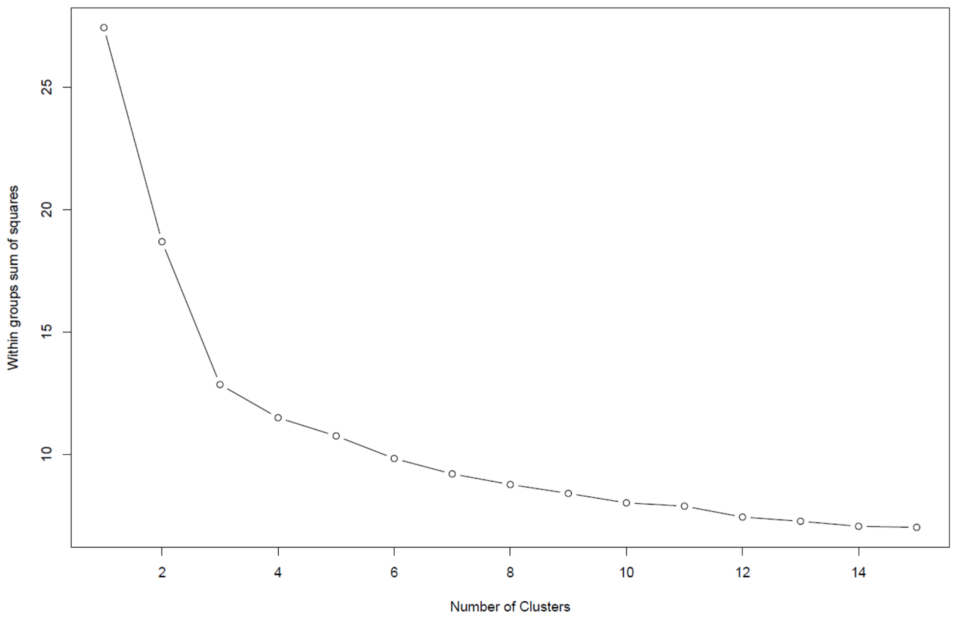

Supplementary Figure S1. Clustering evaluation based on three different approaches. (A) Silhouette analysis. (B) Calinski-Harabasz index. (C) Sum of squares
