# Supplementary material for: Enterotype May Drive the Dietary-Associated Cardiometabolic Risk Factors
Source: Front Cell Infect Microbiol. 2017 Feb 23;7:47. doi: 10.3389/fcimb.2017.00047 (PMC5322172; doi:10.3389/fcimb.2017.00047)
Supplement: Supplementary file 4 [file Image2.PDF]

Supplementary Material

Enterotype may drive the diet-associated cardiometabolic risk factor

Ana Carolina Franco Moraes, Gabriel R. Fernandes, Isis Tande da Silva, Bianca Almeida-Pititto, Everton Padilha Gomes, Alexandre da Costa Pereira, Sandra Roberta G. Ferreira*.

*** Correspondence:** Corresponding Author: sandrafv@usp.br

1 Supplementary Figures and Tables

1.1 Supplementary Figures

(A)

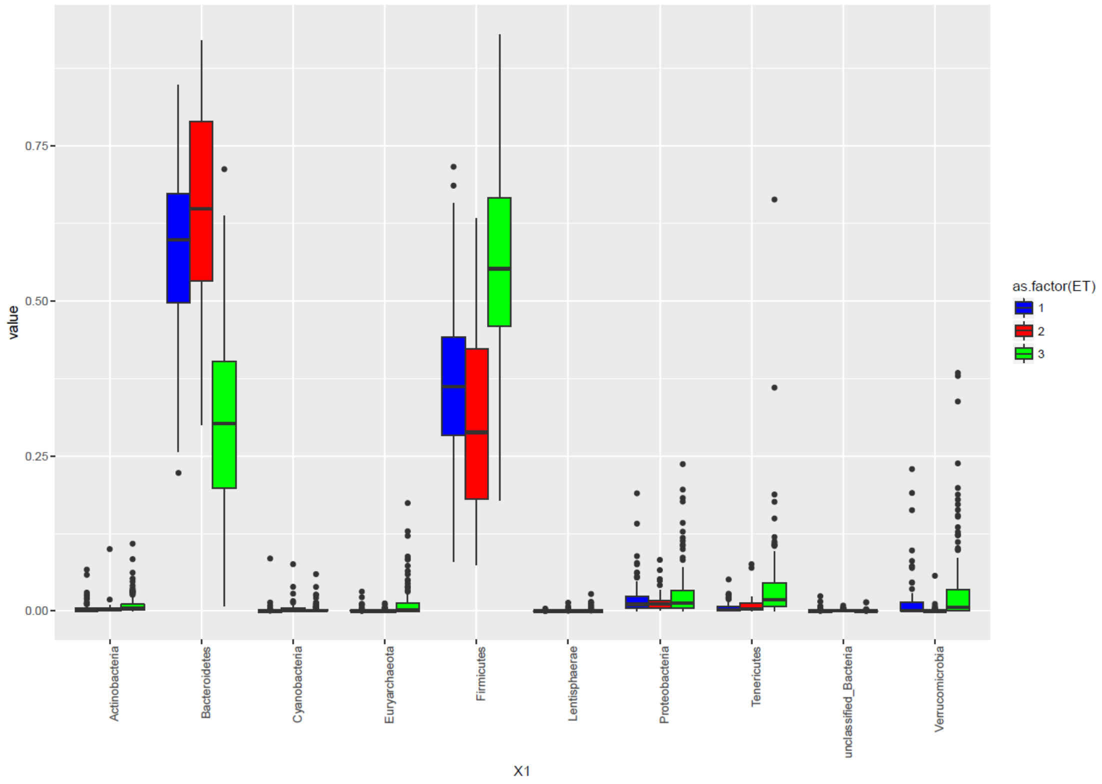

(B)

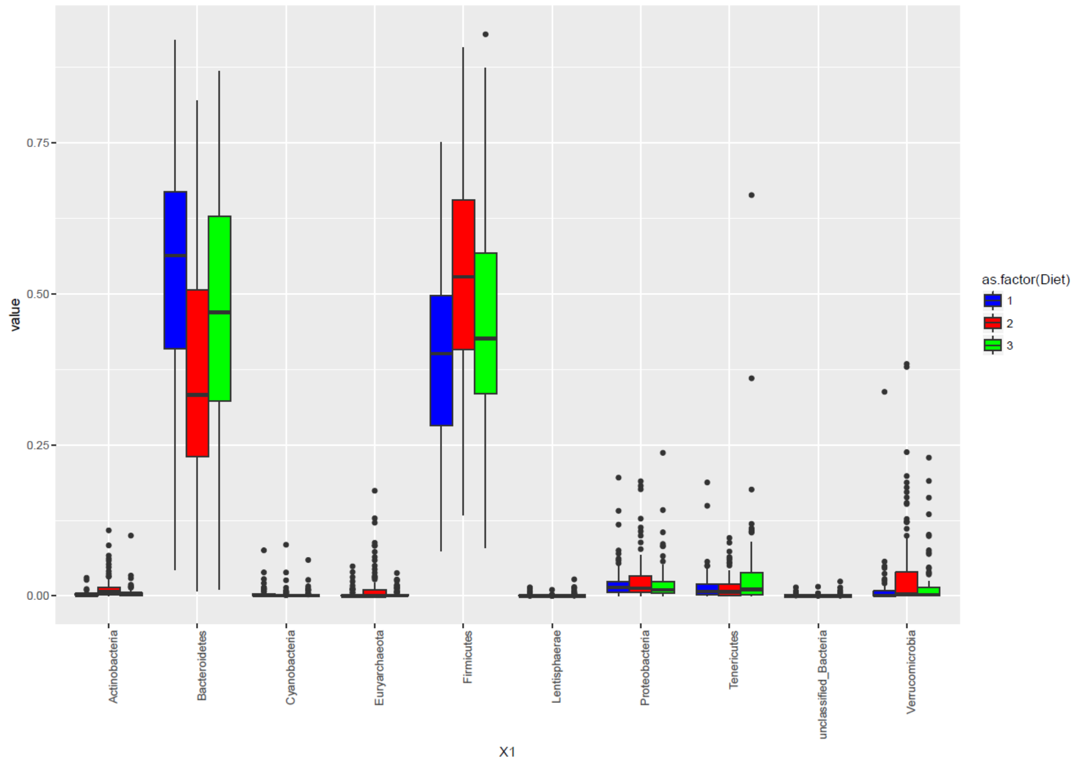
value
0.00
0.25
0.50
0.75
Actinobacteria
Bacteroidetes
Cyanobacteria
Euryarchaeota
Firmicutes
Lentisphaerae
Proteobacteria
Tenericutes
unclassified_Bacteria
Verrucomicrobia
X1
as.factor(Diet)
1
2
3

(C)

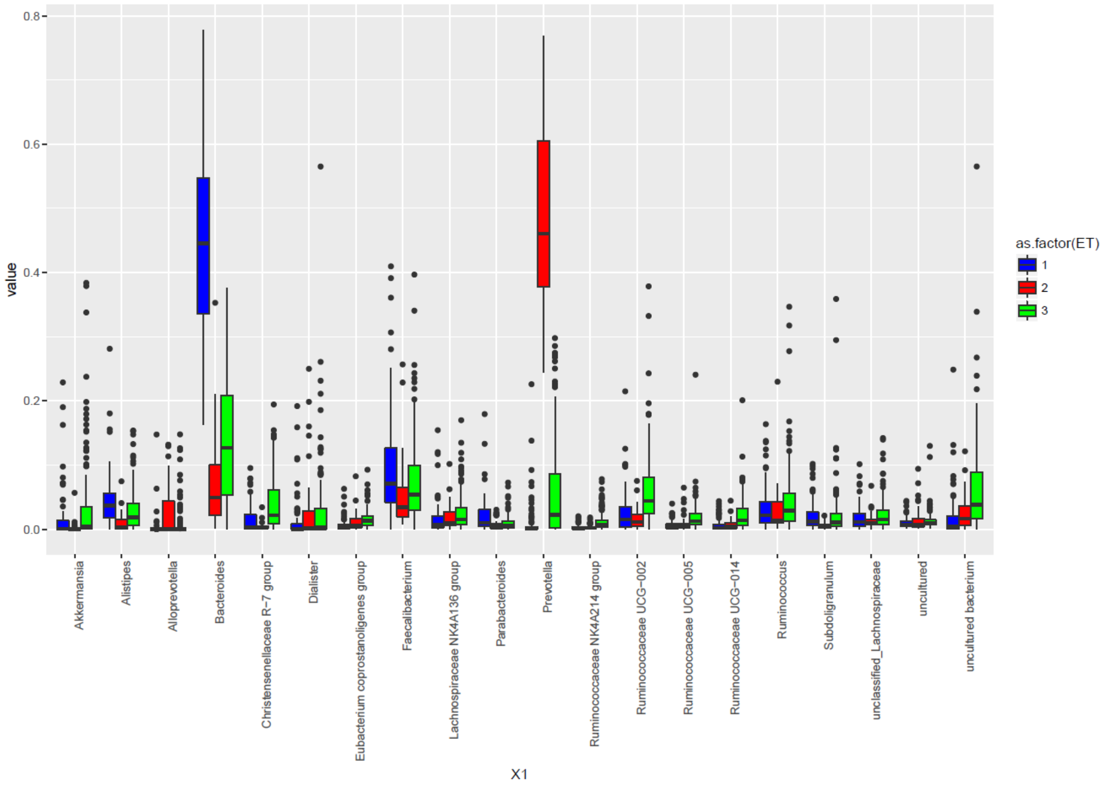
value
0.0
0.2
0.4
0.6
0.8
Akkermansia
Alistipes
Alloprevotella
Bacteroides
Christensenellaceae R−7 group
Dialister
Eubacterium coprostanoligenes group
Faecalibacterium
Lachnospiraceae NK4A136 group
Parabacteroides
Prevotella
Ruminococcaceae NK4A214 group
Ruminococcaceae UCG−002
Ruminococcaceae UCG−005
Ruminococcaceae UCG−014
Ruminococcus
Subdoligranulum
unclassified_Lachnospiraceae
uncultured
uncultured bacterium
X1
as.factor(ET)
1
2
3

(D)

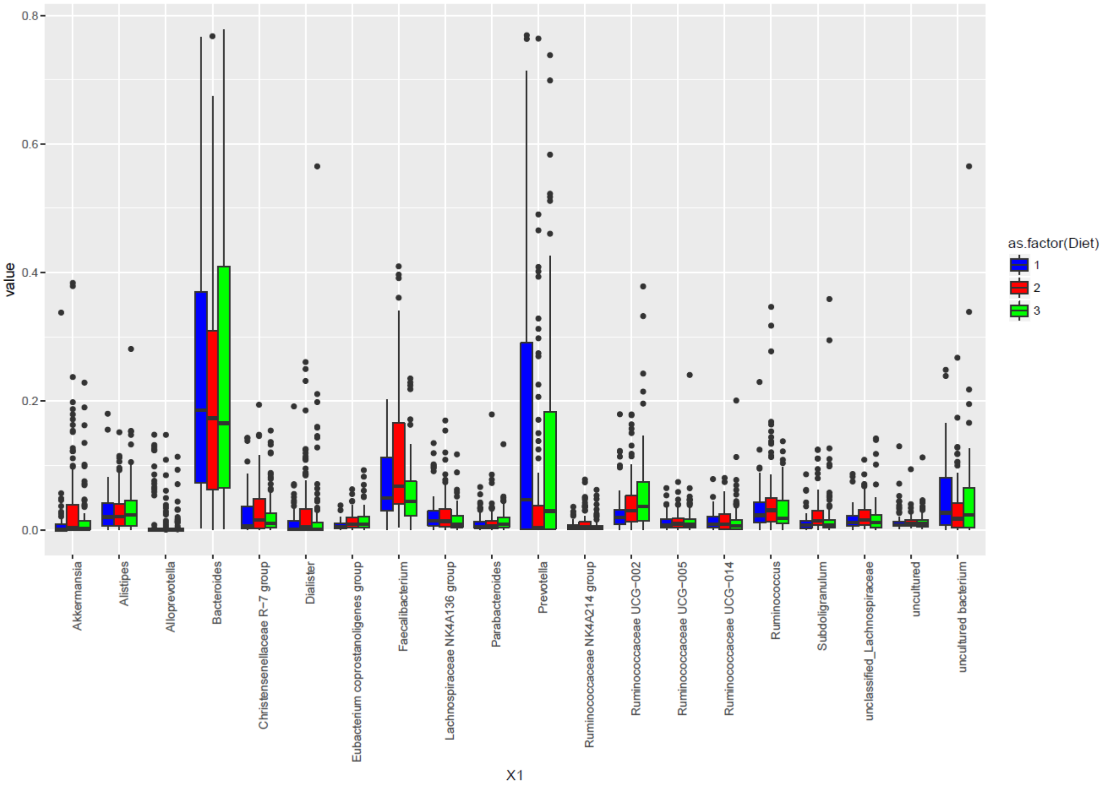

Supplementary Figure S2. **Box plot of the taxonomic relative abundance of the fecal microbiota of 268 participants at the level of phylum (panels A and B) and genus (panels C and D).** The 10 most abundant phyla according to enterotype (A) and to dietary habits classification (B) are shown. The 20 most abundant genera according to enterotype (C) and to dietary habits classification (D) are shown. Boxes represent the interquartile range and the line inside represents the median of relative abundances.
