# Supplementary material for: Enterotype May Drive the Dietary-Associated Cardiometabolic Risk Factors
Source: Front Cell Infect Microbiol. 2017 Feb 23;7:47. doi: 10.3389/fcimb.2017.00047 (PMC5322172; doi:10.3389/fcimb.2017.00047)
Supplement: Supplementary file 5 [file Image3.PDF]

Supplementary Material

Enterotype may drive the diet-associated cardiometabolic risk factor

Ana Carolina Franco de Moraes, Gabriel R. Fernandes, Isis Tande da Silva, Bianca Almeida-Pititto, Everton Padilha Gomes, Alexandre da Costa Pereira, Sandra Roberta G. Ferreira*.

*** Correspondence:** Corresponding Author: sandrafv@usp.br

1 Supplementary Figures and Tables

1.1 Supplementary Figures

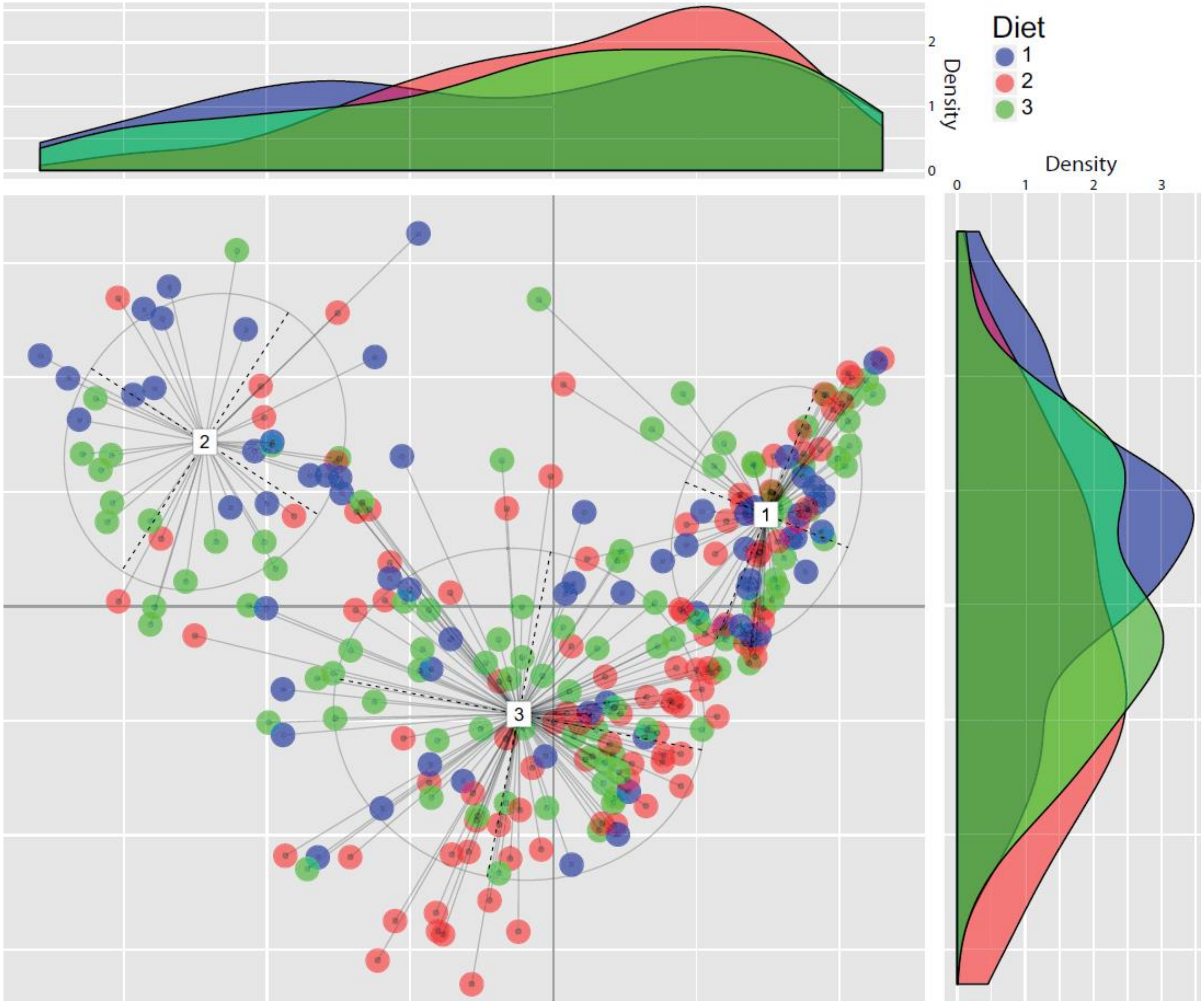

Supplementary Figure S3. Enterotypes identified in 268 participants with different dietary behavior using Principal Coordinate Analysis. Numbers indicate the enterotype they belong to (enterotype 1 is *Bacteroides*, enterotype 2 *Prevotella* and enterotype 3 *Ruminococcaceae*) and colors the dietary behavior (blue is strict vegetarian, red lacto-ovo-vegetarian and green omnivore).
